# Supplementary material for: A Critical View of the Application of the APEX Software (Aqueous Photochemistry of Environmentally-Occurring Xenobiotics) to Predict Photoreaction Kinetics in Surface Freshwaters
Source: Molecules. 2019 Dec 18;25(1):9. doi: 10.3390/molecules25010009 (PMC7017383; doi:10.3390/molecules25010009)
Supplement: Supplementary file 1 [file molecules-25-00009-s001.zip › 3.2.4_gcc-4.4.0.zip!/3.2.4_gcc-4.4.0/3.2.4_gcc-4.4.0/doc/octave/html/interpreter/Increment-Ops.html]

Increment Ops - Untitled


Next: Operator Precedence,
Previous: Assignment Ops,
Up: Expressions


---

### 8.7 Increment Operators

*Increment operators* increase or decrease the value of a variable
by 1. The operator to increment a variable is written as ++. It
may be used to increment a variable either before or after taking its
value.

For example, to pre-increment the variable x, you would write
`++`x. This would add one to x and then return the new
value of x as the result of the expression. It is exactly the
same as the expression x `=` x `+ 1`.

To post-increment a variable x, you would write x`++`.
This adds one to the variable x, but returns the value that
x had prior to incrementing it. For example, if x is equal
to 2, the result of the expression x`++` is 2, and the new
value of x is 3.

For matrix and vector arguments, the increment and decrement operators
work on each element of the operand.

Here is a list of all the increment and decrement expressions.

`++`x: This expression increments the variable x. The value of the expression is the *new* value of x. It is equivalent to the expression x `=` x `+ 1`. `--`x: This expression decrements the variable x. The value of the expression is the *new* value of x. It is equivalent to the expression x `=` x `- 1`. x`++`: This expression causes the variable x to be incremented. The value of the expression is the *old* value of x. x`--`: This expression causes the variable x to be decremented. The value of the expression is the *old* value of x.
